# Potential Biomarkers of Resilience to Microgravity Hazards in Astronauts

**DOI:** 10.7759/cureus.57173

**Published:** 2024-03-29

**Authors:** Piercarlo Minoretti, Giovanni Fortuna, Konstantinos Lavdas, Davide D’Acquino

**Affiliations:** 1 Occupational Health, Studio Minoretti, Oggiono, ITA

**Keywords:** astronauts, microgravity, resilience, biomarkers, spaceflight

## Abstract

Space exploration exposes astronauts to the unique environment of microgravity, which poses significant health challenges. Identifying biomarkers that can predict an individual’s resilience to the stressors of microgravity holds great promise for optimizing astronaut selection and developing personalized countermeasures. This narrative review examines the principal health risks associated with microgravity and explores potential biomarkers indicative of resilience. The biomarkers being evaluated represent a broad spectrum of physiological domains, including musculoskeletal, neurological, immunological, gastrointestinal, cardiovascular, and cutaneous systems. Earth-based microgravity analogs, such as dry immersion and head-down tilt bed rest, may provide valuable platforms to validate candidate biomarkers. However, biomarker sensitivity and specificity must be further evaluated to ensure efficacy and reliability. Establishing a panel of biomarkers predictive of resilience to microgravity-induced health risks would significantly enhance astronaut health and mission success, especially for long-duration exploration missions. Insights gained may also translate to health conditions on Earth characterized by reduced physical activity and mechanical loading.

## Introduction and background

During spaceflight missions, astronauts are exposed to the unique environment of microgravity, which can have profound effects on their health and performance [1]. Microgravity, a condition characterized by the virtual absence of gravitational forces, induces a range of physiological adaptations that can pose significant health risks to crew members [2]. As space agencies plan for longer-duration missions beyond low-Earth orbit, understanding and mitigating the effects of microgravity on astronaut health becomes increasingly critical [3]. While microgravity induces substantial changes in various physiological systems [1, 2], substantial inter-individual variability exists in the extent to which astronauts experience adverse effects from microgravity exposure [4]. Although currently speculative, this suggests that in the future, biomarkers could potentially be used to forecast an individual astronaut's resilience to the challenges presented by microgravity environments [5]. In the context of spaceflight, the identification and monitoring of resilience biomarkers could offer significant benefits [6]. These biomarkers would facilitate the development of personalized countermeasures, tailored to each individual astronaut's unique physiological needs and responses. Moreover, resilience biomarkers could serve as leading indicators of an astronaut's health status during missions.

**Figure 1:**
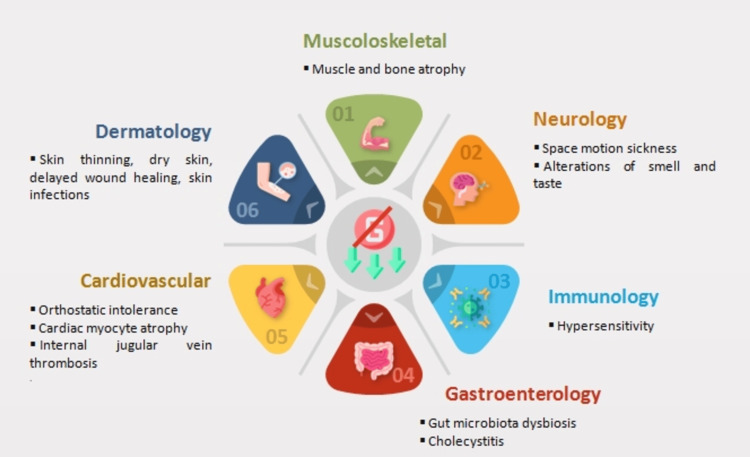
Main effects of microgravity on different physiological domains Image credit: Piercarlo Minoretti

The concept of developing biomarkers to predict performance in microgravity environments has major translational implications. However, to our knowledge, no comprehensive review has been conducted on potential biomarkers of resilience to the specific challenges posed by microgravity. This manuscript aims to address this gap by summarizing the major health risks associated with microgravity exposure and proposing candidate biomarkers that may predict resilience to these risks based on terrestrial studies. We specifically focused on biomarkers that can be measured prior to spaceflight to assess baseline status, with the future goal of building a predictive model of an astronaut’s resilience to the physiological adaptations induced by microgravity. The biomarkers being evaluated represent a broad spectrum of physiological domains, including musculoskeletal, neurological, immunological, gastrointestinal, cardiovascular, and cutaneous systems (Figure 1).

## Review

Search strategy

To identify relevant references for this review, a comprehensive search was conducted in the PubMed database for peer-reviewed articles published in English between January 1, 2013, and December 31, 2023. The search strategy employed a combination of key terms related to the effects of microgravity on various physiological systems. These terms were used in combination with "astronauts" to ensure the results were specific to the astronaut population. The search query was constructed as follows: ("microgravity") AND "astronauts" AND ("musculoskeletal" OR "neurology" OR "immunology" OR "gastroenterology" OR "cardiovascular" OR "dermatology" OR "biomarkers" OR "resilience"). This search strategy yielded a total of 842 articles. The search results were then screened for relevance to the specific topics addressed in this review. After this initial screening, the bibliographies of the selected articles were manually reviewed to identify any additional relevant studies not captured by the primary search. The final reference list included a total of 76 articles that were deemed most relevant to the topics covered in this review. These articles form the basis of the evidence presented and discussed throughout the manuscript.

Musculoskeletal resilience biomarkers

In the absence of Earth’s gravity, the musculoskeletal system experiences reduced mechanical loading, resulting in adaptive changes [7]. Muscle atrophy occurs rapidly, with up to 20% loss of muscle mass, particularly in postural and anti-gravity muscles [8]. This is accompanied by a shift from slow-twitch to fast-twitch muscle fibers [9]. Atrophy impairs strength, physical performance, and the ability to perform mission-critical tasks [10]. Bone loss occurs at a rate of 0.5−1.5% per month in weight-bearing bones such as the hip and spine [11]. This is due to increased bone resorption and decreased bone formation, leading to reduced bone mineral density and altered bone structure [12]. Notably, astronauts are at risk for fractures and early-onset osteoporosis [13].

Potential resilience biomarkers for these conditions include low baseline levels of myostatin, a protein that inhibits muscle growth, which may indicate a reduced tendency for muscle atrophy [14]. Myostatin, a member of the transforming growth factor-β superfamily, is a negative regulator of skeletal muscle mass [15]. Increased myostatin signaling has been implicated in the muscle atrophy that occurs during exposure to microgravity in mice [16]. Inhibition of myostatin using neutralizing antibodies or other agents has shown promise in mitigating microgravity-induced muscle loss in animal models [17]. In mice exposed to microgravity during spaceflight, myostatin inhibition using an anti-myostatin antibody significantly increased muscle mass compared to untreated mice [17]. The degree of muscle hypertrophy varied between different muscle groups, with some responding equally well in the presence or absence of gravity. Notably, myostatin inhibition also modulated the expression of genes involved in muscle architecture that were altered by microgravity exposure [17]. Elevated levels of insulin-like growth factor-1 (IGF-1), a hormone associated with muscle and bone growth [18], may also serve as a resilience biomarker. IGF-1 has been shown to stimulate myogenesis via protein synthesis and reduce muscle degeneration [19]. It also increases the proliferative capacity of muscle satellite cells [20]. In addition, IGF-1 production increases in the satellite cells of damaged muscles, promoting their growth and differentiation into muscle cells [19]. Mechanical loading affects skeletal muscle production of IGF-1 [18], and low IGF-1 levels are associated with reduced handgrip strength and poor physical performance [18, 20]. IGF-1 is potentially useful in the management of muscle atrophy [18] and plays a key role in bone metabolism, stimulating bone formation and remodeling [21]. Strategies to maintain or increase IGF-1 levels, such as exercise and nutritional interventions [22], may help counteract microgravity-induced musculoskeletal changes.

Central nervous system resilience biomarkers

Microgravity exposure during spaceflight can have profound effects on the central nervous system (CNS), leading to a range of potential neurological issues [23, 24]. One of the most common CNS disturbances experienced by astronauts is space motion sickness (SMS), which is characterized by symptoms such as nausea, vomiting, headache, and disorientation [25, 26]. SMS is thought to result from a sensory conflict between visual, vestibular, and proprioceptive inputs in the microgravity environment [27]. Potential biomarkers for resilience against SMS may involve a blunted increase in plasma arginine vasopressin (AVP) levels after rotation [28]. AVP is a hormone that plays a role in fluid balance and has been implicated in the development of motion sickness [29]. Studies have shown that individuals with a smaller increase in AVP levels after exposure to provocative motion stimuli are less susceptible to motion sickness [30]. Therefore, assessing baseline AVP levels and the magnitude of AVP response to vestibular stimulation could help identify astronauts who are more resilient to SMS. Microgravity can also lead to alterations in smell and taste perception [31], which can have significant effects on food intake, nutrition, and overall well-being. The underlying mechanisms of these sensory changes are not fully understood but may involve structural and functional adaptations in the olfactory and gustatory systems [31, 32]. High levels of sonic hedgehog (Shh), a protein involved in the development and maintenance of sensory neurons, in nasal mucus and saliva could indicate resilience to alterations in smell and taste, respectively [33, 34]. Shh plays a critical role in the regeneration and plasticity of olfactory and gustatory receptors [35]. Astronauts with higher baseline levels of Shh in these fluids may be more capable of adapting to the sensory challenges posed by microgravity.

Immune system resilience biomarkers

Microgravity exposure during spaceflight can have significant effects on the immune system, leading to a range of alterations in immune function and increased susceptibility to infections [36, 37]. One of the potential consequences of microgravity-induced immune dysregulation is the development of hypersensitivity reactions [37]. Hypersensitivity reactions are exaggerated or inappropriate immune responses to specific antigens, which can manifest as allergic reactions, autoimmune disorders, or other inflammatory conditions [38, 39]. In the context of spaceflight, hypersensitivity reactions may be triggered by factors such as altered antigen presentation, changes in cytokine profiles, and increased oxidative stress [36]. Eosinophils, a type of white blood cell, are involved in the initiation and maintenance of allergic inflammation through the release of various mediators, such as cytokines, chemokines, and granule proteins. Elevated eosinophil counts are often associated with allergic diseases, such as asthma, allergic rhinitis, and atopic dermatitis [40]. Therefore, maintaining normal ranges of eosinophils could indicate resilience against hypersensitivity reactions in the microgravity environment. Astronauts with lower baseline eosinophil counts and a reduced propensity for eosinophil activation may be less susceptible to developing allergic reactions during spaceflight. Immunoglobulin E (IgE) is another key player in the development of hypersensitivity reactions. Upon exposure to an allergen, IgE binds to the allergen and triggers the release of inflammatory mediators from mast cells and basophils, leading to the manifestation of allergic symptoms [41]. Elevated IgE levels are a hallmark of atopic disorders and are often used as a biomarker for allergic sensitization [41]. Hence, monitoring IgE levels before and after spaceflight could help identify individuals who are at a higher risk of experiencing allergic reactions in the microgravity environment. Periostin, a matricellular protein, has recently emerged as a promising biomarker for allergic inflammation [42]. Periostin has been implicated in the pathogenesis of allergic diseases, such as asthma and atopic dermatitis [43]. Elevated serum periostin levels have been associated with increased disease severity and resistance to treatment in patients with allergic disorders [42, 43]. In the context of spaceflight, astronauts with lower baseline periostin levels and a reduced propensity for periostin upregulation in response to inflammatory stimuli may be less susceptible to developing allergic reactions during long-duration space missions.

Gastrointestinal system resilience biomarkers

Microgravity exposure during spaceflight can have profound effects on the gastrointestinal (GI) system, leading to alterations in gut microbiota composition [44, 45] and increased intestinal permeability [46]. One of the major concerns is the development of gut microbiota dysbiosis, which refers to an imbalance in the composition and function of the gut microbial community [47]. Dysbiosis has been associated with various GI disorders, such as inflammatory bowel disease and irritable bowel syndrome [48]. In the context of spaceflight, factors such as altered nutrient intake, stress, and radiation exposure can interact with microgravity to promote the development of gut microbiota dysbiosis [49]. Serum levels of gut permeability biomarkers, such as zonulin and lipopolysaccharide (LPS) [50], may serve as potential indicators of resilience against microgravity-induced gut microbiota dysbiosis. Zonulin is a protein that regulates intestinal permeability by modulating the tight junctions between epithelial cells [51]. Elevated serum zonulin levels have been associated with increased gut permeability and the development of various GI disorders [52]. LPS, a component of the outer membrane of gram-negative bacteria, is another marker of gut permeability [53]. When the intestinal barrier is compromised, LPS can translocate from the gut lumen into the systemic circulation, leading to endotoxemia and inflammation [53]. Astronauts with normal serum levels of zonulin and LPS may be more resilient to the development of gut microbiota dysbiosis and its associated complications during spaceflight. Plasma levels of short-chain fatty acids (SCFAs), such as acetate, propionate, and butyrate [54], may also serve as biomarkers of gut microbiota health and resilience against microgravity-induced dysbiosis. SCFAs are produced by the fermentation of dietary fibers by the gut microbiota and play a crucial role in maintaining intestinal homeostasis [55]. They exert various beneficial effects, such as regulating intestinal motility, promoting the growth of beneficial bacteria, and modulating immune responses [54]. Reduced plasma levels of SCFAs have been associated with gut microbiota dysbiosis and the development of GI disorders [56]. Astronauts with normal plasma levels of SCFAs may have a more resilient gut microbiota that is better equipped to withstand the challenges posed by the microgravity environment. Another potential GI issue during spaceflight is the development of cholecystitis [57]. Normal biochemical markers of cholestasis and liver injury, such as alkaline phosphatase, gamma-glutamyl transferase, and bilirubin levels, along with normal abdominal ultrasound findings, may indicate resilience against the development of cholecystitis during spaceflight.

Cardiovascular system resilience biomarkers

Microgravity exposure during spaceflight induces a constellation of cardiovascular adaptations that can lead to orthostatic intolerance [58] and cardiac deconditioning [59]. Orthostatic intolerance, characterized by an inability to maintain blood pressure and cerebral perfusion upon assuming an upright posture, affects up to 80% of astronauts returning from long-duration missions [60]. The mechanisms underlying post-spaceflight orthostatic intolerance are multifactorial and include hypovolemia, altered autonomic control, and reduced vascular responsiveness [61]. Potential biomarkers for resilience against orthostatic intolerance include normal heart rate variability (HRV) and normal circulating levels of renin, aldosterone, and norepinephrine. HRV, a measure of the beat-to-beat variations in heart rate, reflects the autonomic control of the cardiovascular system [62]. Maintained HRV during simulated microgravity may indicate a preserved ability to regulate blood pressure and cardiac output upon return to Earth's gravity. Similarly, astronauts who maintain their blood volume and exhibit normal levels of renin, aldosterone, and norepinephrine, key regulators of fluid balance and vascular tone [63], may be less susceptible to post-flight orthostatic intolerance. Cardiac myocyte atrophy, characterized by a reduction in cardiomyocyte size and contractile function, is another consequence of prolonged microgravity exposure [64]. This atrophic remodeling is thought to result from altered cardiac loading conditions and reduced metabolic demands in the microgravity environment [65]. The absence of mutations in the phosphatidylinositol-4,5-bisphosphate 3-kinase catalytic subunit alpha (*PIK3CA*) gene and the presence of a “*PTEN*-less” phenotype - characterized by loss or inactivation of the phosphatase and TENsin homolog deleted on chromosome 10 (*PTEN*) gene - may contribute to the maintenance of cardiac myocyte structure and function, potentially serving as biomarkers for resilience against cardiac myocyte atrophy. The *PIK3CA* gene encodes the p110α catalytic subunit of phosphatidylinositol 3-kinase α (PI3Kα), which is a lipid kinase that plays a crucial role in cell growth, survival, and motility [66]. In the context of the heart, PI3Kα has been shown to be protective in various models of heart failure and cardiac stress [66]. Specifically, PI3Kα activation is part of a compensatory response during heart failure, and reduced PI3Kα activity has been observed in human and dog hearts with dilated cardiomyopathy [66]. Moreover, PI3Kα deficiency in mice leads to accelerated progression of heart failure and exacerbates cardiac atrophy [66]. This suggests that normal PI3Kα activity, which would be altered by *PIK3CA* mutations, is important for maintaining cardiac structure and function under stress. *PTEN* is a negative regulator of the PI3Kα pathway, and its loss has been shown to provide marked and persistent protection against cardiac stress [67]. *PTEN* loss or inactivation, referred to as “PTEN-less”, has been implicated in the regulation of cardiac physiological and pathological processes, including hypertrophy, proliferation, apoptosis, and survival [67]. Inactivation of *PTEN* in mouse models has been shown to protect the heart from hypertrophic growth in pathological remodeling [67]. Given the protective roles of PI3Kα activity [66] and the negative regulation by *PTEN* in the heart [67], it is plausible that the absence of PIK3CA mutations and the “*PTEN*-less” phenotype could be indicative of a heart’s resilience to microgravity-induced atrophy. The absence of *PIK3CA *mutations would suggest normal PI3Kα activity, which is necessary for a resilient actin cytoskeleton and compensatory responses during heart failure [66]. Similarly, the presence of a “*PTEN*-less” phenotype would ensure proper regulation of the PI3K pathway, which is involved in cardiomyocyte survival and function [67]. Therefore, astronauts with normal *PIK3CA* and the “*PTEN*-less” phenotype may be less prone to microgravity-induced cardiac atrophy. Another significant cardiovascular risk associated with microgravity is the development of internal jugular vein thrombosis (IJVT) [68]. IJVT is a potentially serious condition that can lead to pulmonary embolism and other complications [69]. The risk of IJVT is increased in the microgravity environment due to factors such as fluid shifts, venous stasis, and altered coagulation pathways [70]. Normal levels of D-dimer, prothrombin time, partial thromboplastin time, platelet count, and fibrinogen, along with negative Factor V Leiden and prothrombin G20210A mutation [71, 72], could suggest a lower risk of IJVT. These biomarkers reflect the balance between coagulation and fibrinolysis and can help identify individuals with a more favorable hemostatic profile [71, 72]. Astronauts with normal levels of these markers and an absence of prothrombotic single nucleotide polymorphisms may be more resilient to the development of IJVT during spaceflight.

Cutaneous system resilience biomarkers

Astronauts often experience skin-related issues during space missions, including skin thinning, dryness, slower wound healing, and a heightened risk of infections [73]. Recent research has identified biomarkers that could signal skin resilience in space. Among these, matrix metalloproteinases (MMPs) have drawn significant interest. A study examining the impact of simulated microgravity on human mesenchymal stem cells found that such conditions trigger proteolytic activity within the cellular matrix and reduce the cells' adherence to the extracellular matrix [74]. Consequently, astronauts with naturally lower levels of MMPs might be more resistant to the skin damage and wound healing challenges posed by microgravity. In addition to biochemical markers, skin biophysical parameters could also serve as indicators of resilience. These parameters include skin hydration, transepidermal water loss (TEWL), pH, and elasticity [75]. Astronauts with well-maintained skin barrier function, as evidenced by optimal hydration levels, low TEWL, slightly acidic pH, and good elasticity, may be less susceptible to the adverse effects of microgravity on the skin. To elucidate the role of these biomarkers in predicting skin resilience, longitudinal studies involving astronauts are necessary. Pre-flight assessments of MMPs and skin biophysical parameters should be conducted and compared with post-flight measurements. Additionally, correlations between these biomarkers and the incidence and severity of dermatological issues during spaceflight should be investigated.

Discussion

This review has provided a summary of potential biomarkers that may predict resilience to microgravity hazards across multiple physiological systems (Table 1).

**Table 1:** Overview of potential biomarkers for resilience against the adverse effects of microgravity

Microgravity-related Hazards	Alterations	Candidate Resilience Biomarker
Musculoskeletal		
	Muscle and bone atrophy	Low baseline myostatin levels, elevated insulin-like growth factor-1 levels
Neurology		
	Space motion sickness	Blunted increase in plasma arginine vasopressin levels after rotation
	Altered smell and taste	Smell: high sonic hedgehog levels in nasal mucus; Taste: high sonic hedgehog levels in saliva
Immunology		
	Hypersensitivity	Eosinophils, IgE levels, and periostin within the normal range
Gastroenterology		
	Gut microbiota dysbiosis	Normal serum levels of gut permeability biomarkers (zonulin, lipopolysaccharide); normal plasma short-chain fatty acid levels
	Cholecystitis	Normal biochemical markers of cholestasis and liver injury, normal findings on abdominal ultrasound
Cardiovascular		
	Orthostatic intolerance	Normal heart rate variability, blood volume, and blood pressure. Renin, aldosterone, and norepinephrine within the normal range
	Cardiac myocyte atrophy	Absence of *PIK3CA* mutations and "*PTEN*-less" phenotype
	Internal jugular vein thrombosis	Normal levels of D-dimer, normal prothrombin time and partial thromboplastin time, normal platelet count, normal fibrinogen, no carriage of prothrombotic single nucleotide polymorphisms
Dermatology		
	Skin thinning, dryness, delayed wound healing, cutaneous infections	Low baseline matrix metalloproteinases levels; normal skin biophysical parameters

A carefully selected panel of biomarkers, measured at baseline and in response to microgravity, could be used to comprehensively assess an astronaut’s overall resilience profile. This personalized approach would enable tailored countermeasures to mitigate individual risks. Insights gained may also translate to health conditions on Earth characterized by reduced physical activity and mechanical loading. Earth-based microgravity analogs, such as dry immersion and head-down tilt bed rest [76], may provide valuable platforms to validate candidate resilience biomarkers in a controlled setting. Unfortunately, the relative contribution of each biomarker to overall resilience and the potential interactions between biomarkers across physiological systems are still unclear. Albeit not currently implemented, standardized protocols for biomarker measurement and interpretation need to be established and harmonized across space agencies. Despite these hurdles, the development and validation of a robust panel of resilience biomarkers has the potential to revolutionize astronaut selection, monitoring, and individualized countermeasure regimens. As space agencies prepare for ambitious exploration missions to the Moon, Mars, and beyond, investing in this critical biomarker research will be essential for ensuring crew health and mission success in the face of the unforgiving spaceflight environment. Future research should focus on conducting prospective studies to validate candidate biomarkers, developing integrated predictive models that account for biomarker interactions, and establishing consensus protocols for resilience biomarker testing. Emerging technologies such as organ-on-a-chip systems and artificial intelligence-driven data analysis may accelerate progress. Importantly, research should also investigate the potential to enhance resilience through targeted interventions, guided by an individual's biomarker profile.

## Conclusions

The dawn of personalized medicine for spaceflight is on the horizon. By harnessing the power of resilience biomarkers, we can unlock the full potential of human adaptability and usher in a new era of space exploration. The stars are calling - and with rigorous research to develop the most suitable biomarkers, we can ensure that future astronauts are equipped to answer that call as safely and successfully as possible.
